# Production and organization of neocortical interneurons

**DOI:** 10.3389/fncel.2013.00221

**Published:** 2013-11-21

**Authors:** Khadeejah T. Sultan, Keith N. Brown, Song-Hai Shi

**Affiliations:** ^1^Developmental Biology Program, Memorial Sloan-Kettering Cancer CenterNew York, NY, USA; ^2^Graduate Program in Neuroscience, Weill Cornell Medical CollegeNew York, NY, USA

**Keywords:** neocortex, inhibition, interneurons, neurogenesis, migration, organization

## Abstract

Inhibitory GABA (γ-aminobutyric acid)-ergic interneurons are a vital component of the neocortex responsible for shaping its output through a variety of inhibitions. Consisting of many flavors, interneuron subtypes are predominantly defined by their morphological, physiological, and neurochemical properties that help to determine their functional role within the neocortex. During development, these cells are born in the subpallium where they then tangentially migrate over long distances before being radially positioned to their final location in the cortical laminae. As development progresses into adolescence, these cells mature and form chemical and electrical connections with both glutamatergic excitatory neurons and other interneurons ultimately establishing the cortical network. The production, migration, and organization of these cells are determined by vast array of extrinsic and intrinsic factors that work in concert in order to assemble a proper functioning cortical inhibitory network. Failure of these cells to undergo these processes results in abnormal positioning and cortical function. In humans, this can bring about several neurological disorders including schizophrenia, epilepsy, and autism spectrum disorders. In this article, we will review previous literature that has revealed the framework for interneuron neurogenesis and migratory behavior as well as discuss recent findings that aim to elucidate the spatial and functional organization of interneurons within the neocortex.

## INTRODUCTION

The telencephalon is an extremely complex biological entity and is responsible for the higher brain functions of the central nervous system. Located in the roof of the dorsal telencephalon (pallium), the neocortex is the largest and most pivotal structure of the mammalian telencephalon playing a critical role in numerous processes such as cognition and sensory perception (Rakic, 1988; Leingartner et al., 2007; O’Leary and Sahara, 2008; Tomassy et al., 2010). These functions are carried out in regionally distinct areas each of which are typically arranged in six layers (lamina), which differ in neuronal composition, density, and connectivity. Conversely, the cortical microcircuitry is thought to be functionally organized into vertically arrayed radial units or columns that span the cortical layers and consist of two major classes of neurons: glutamatergic excitatory cells (pyramidal and spiny stellate neurons) and GABA (γ-aminobutyric acid)-ergic inhibitory interneurons (Hensch, 2005). Glutamatergic excitatory neurons comprise the majority cells in the neocortex and project their axons long distances generating the output both within the cortex and to distant brain regions. GABAergic interneurons are local circuit cells responsible for inhibitory transmission in the neocortex. While they only comprise approximately 20% of the neocortical milieu, interneurons play a key role in modulating cortical output and plasticity through a rich variety of inhibitions made possible by an assortment of distinct subtypes characterized by their morphological, physiological, and neurochemical properties (Markram et al., 2004; Huang et al., 2007; Ascoli et al., 2008; DeFelipe et al., 2013).

During cortical development, glutamatergic excitatory neurons are generated in the ventricular zone of the developing dorsal telencephalon and migrate radially into the cortical plate (CP), where as neocortical interneurons are produced in the developing ventral telencephalon (subpallium) and migrate tangentially over long distances to reach their destination in the neocortex (Rakic, 1978; Anderson et al., 2001, 2002; Molyneaux et al., 2007; Rakic, 2007). Previous genetic and transplantation studies have demonstrated that distinct interneuron subtypes are produced in spatially and temporally distinct regions in the subpallium (Xu et al., 2004; Butt et al., 2005; Flames et al., 2007; Fogarty et al., 2007; Miyoshi et al., 2007; Wonders et al., 2008; Xu et al., 2008; Miyoshi et al., 2010). Proper development and functioning of the neocortex critically depends on the coordinated production and migration of excitatory and inhibitory neurons (Parnavelas, 2002; Powell et al., 2003a; Kowalczyk et al., 2009; Bedogni et al., 2010; Lodato et al., 2011a; Bartolini et al., 2013). To this point, disruption of the developing GABAergic neocortical inhibitory network has been implicated in several neurological disorders in humans, including schizophrenia, epilepsy, and autism (Lewis, 2000; Armijo et al., 2002; Rubenstein and Merzenich, 2003; Powell et al., 2003a; Levy and Degnan, 2013). It is, therefore, absolutely critical to gain a more detailed understanding of the rules governing interneuron development and how these processes result in the formation of the neocortical inhibitory circuitry.

## INTERNEURON DIVERSITY

**FIGURE 1:**
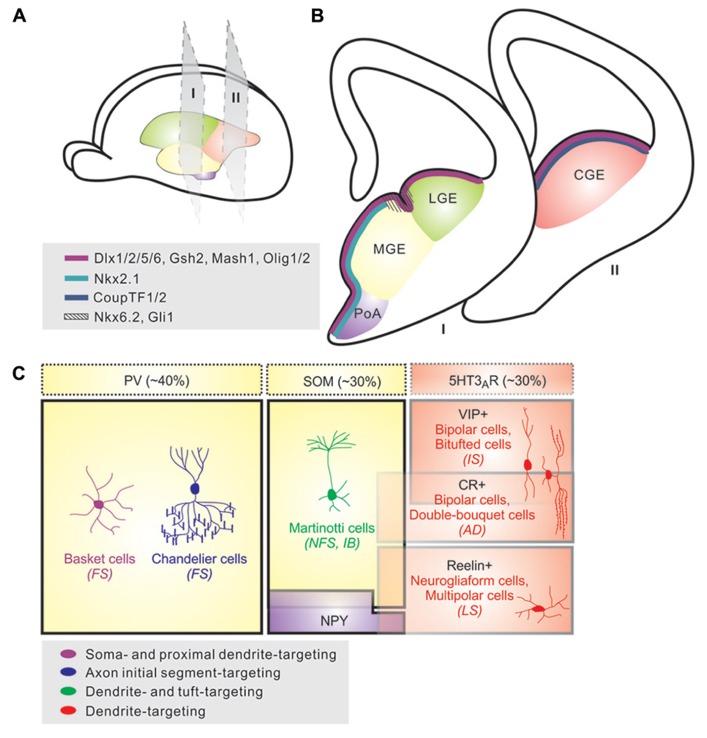
**Origins and diversity of neocortical interneurons.** **(A)** Neocortical interneurons are derived from progenitor cells located in the proliferative zones of the ventral telencephalon, specifically within the medial ganglionic eminence (MGE) and caudal ganglionic eminence (CGE). A small proportion is produced in the preoptic area (PoA). **(B)** Various transcription factors are expressed in distinct patterns throughout the saubpallial germinal zones; whereas Dlx1/2/5/6, Gsh2, Mash1, and Olig1/2 (magenta) are expressed in subventricular zone of the entire GE region, transcription factors such as Nkx2.1 (light blue) and CoupTF1/2 (dark blue) are expressed specifically within the MGE/preoptic area (PoA) and CGE, respectively. Nkx6.2 and Gli1 (hashed lines) display a restricted expression pattern in the sulcus region between MGE and LGE. **(C).** Neocortical interneurons are highly diverse and can be defined based on morphology, neurochemical expression, electrophysiological properties, and subcellular synaptic targeting specificity. About 40% of neocortical interneurons exhibit fast-spiking (FS) electrophysiological profiles, and are comprised of basket and chandelier cells; these cells largely express parvalbumin (PV), although some chandelier cells are PV-negative. Cells expressing somatostatin (SOM) account for ~30% of the neocortical interneurons that are morphologically heterogeneous (e.g., Martinotti cells) and typically exhibit non-FS physiological characteristics. The remaining ~30% of neocortical interneurons largely express the 5-hydroxytryptamine 3A receptor (5-HT3AR) and are comprised of vasointestinal peptide (VIP)-expressing and/or calretinin (CR)-expressing cells with bipolar or double-bouquet morphologies and fast adapting firing (AD) patterns, as well a group of Reelin-expressing, late-spiking (LS), neurogliaform cells. Additionally, a small population of cortical interneurons consists of multipolar cells that contain NPY and display irregular or fast AD firing properties.

Once referred to as “short-axon” neurons by Ramon y Cajal, GABAergic interneurons are key regulators of cortical activity. They are classified by their dendritic and axonal arborization, firing properties, synaptic targets, and immunohistochemical content (**Figure 1**; Monyer and Markram, 2004; Ascoli et al., 2008; Rudy et al., 2011; DeFelipe et al., 2013). Each of these properties influences each interneuron’s specific role within the cortical circuitry. Current data suggests that ~40% of neocortical interneurons exhibit fast-spiking electrophysiological profiles, and are comprised of basket and chandelier cells; these cells largely express the cytoplasmic calcium binding protein parvalbumin (PV), although some chandelier cells are PV-negative (Markram et al., 2004; Taniguchi et al., 2012). Cells expressing the neuropeptide somatostatin (SOM) account for ~30% of the neocortical interneurons that are morphologically heterogeneous and typically exhibit non-fast spiking physiological characteristics (Ma et al., 2006; McGarry et al., 2010; Xu et al., 2013). The remaining ~30% of neocortical interneurons largely express the 5-hydroxytryptamine (serotonin) receptor 3A (5-HT3AR) and are comprised of vasoactive intestinal peptide (VIP)-expressing and/or calretinin (CR)-expressing cells with bipolar or double-bouquet morphologies and rapidly adapting firing patterns, as well a group of reelin-expressing, late-spiking (LS), neurogliaform cells (Lee et al., 2010; Rudy et al., 2010; Armstrong et al., 2012; Ma et al., 2013). Additionally, a small population of cortical interneurons consists of multipolar cells that contain neuropeptide Y (NPY) and display irregular or rapidly adapting firing properties (Lee et al., 2010). Other molecular markers such as Kv3.1, cholecystokinin (CCK), and neuronal nitric oxide synthase (nNOS) are good indicators of subtype identity while others such as calbindin (CB) and Kv3.2 are expressed in a variety of cell types (DeFelipe et al., 1993; Kubota and Kawaguchi, 1994; Cauli et al., 1997; Gonchar and Burkhalter, 1997; Kubota and Kawaguchi, 1997; Chow et al., 1999; Garaschuk et al., 2000; Gupta et al., 2000; Monyer and Markram, 2004). While this classification system is largely accepted by the field, many researchers recognize it as a work in progress. This is because distinct interneuron subtypes often have one or more overlapping characteristics with other subtypes, which has led some to question whether interneuron diversity should be considered, at least to some degree, on a continuum as opposed to more precisely defined subtypes. Efforts are currently underway to further classify subtypes based on their genomic profile and additional protein markers.

## NEUROGENESIS OF NEOCORTICAL INTERNEURONS

Several fate-mapping and transplantation studies in rodents have identified the ventral telencephalon (subpallium) as the sole source for all neocortical interneurons (Xu et al., 2004; Butt et al., 2005; Flames et al., 2007; Fogarty et al., 2007; Miyoshi et al., 2007; Wonders et al., 2008; Xu et al., 2008; **Figures 1A,B**). Much like the production of glutamatergic neurons in the dorsal telencephalon, neocortical interneuron neurogenesis occurs proximal to the ventricle of the developing neuroepithelium, with the majority of neocortical interneurons produced between embryonic days 11–17 (E11–E17). Newborn GABAergic cells tangentially migrate over long distances from the subpallium to the cortex where they integrate in an “inside-out” pattern where earlier born interneurons occupy deeper cortical lamina than their more superficial cohorts (Faux et al., 2012). Interestingly, interneurons and projection neurons born at the same time often reside in the same cortical layers suggesting some degree of coordination between these two processes (Anderson et al., 1997; Tamamaki et al., 1997; Parnavelas, 2000; Marin and Rubenstein, 2001; Lodato et al., 2011a).

During embryonic development, the ventral telencephalon consists of the ganglionic eminences (GE) and preoptic area (PoA)/ anterior entopeduncular (AEP) domains. The GE can be further subdivided into three anatomically distinct regions namely the medial (MGE), lateral (LGE), and caudal (CGE) ganglionic eminences. The MGE and CGE, together with the AEP/PoA, which is located close to the telencephalic stalk in the subpallial domain, are the sole source of cortical interneurons in rodents (Wonders and Anderson, 2006; Batista-Brito and Fishell, 2009; Gelman and Marin, 2010; Welagen and Anderson, 2011). As embryonic development concludes, the morphological boundaries between these regions recede and are no longer recognizable in the post-natal brain.

**FIGURE 2:**
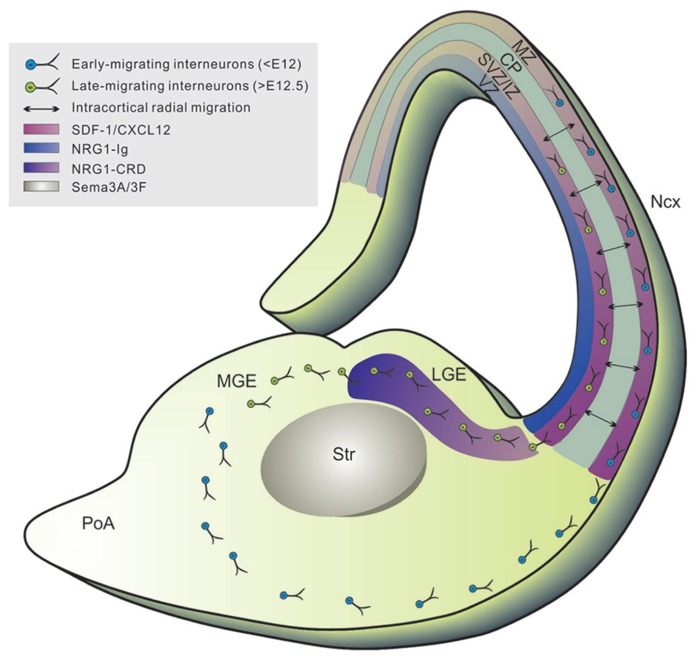
**Tangential migration of neocortical interneurons.** Newborn neocortical interneurons follow two tangentially oriented migratory streams to enter the cortex: a superficially migrating early cohort (blue) migrates through the marginal zone (MZ), and a deeply migrating second and more prominent cohort (green) migrates through the lower intermediate zone (IZ) and subventricular zone (SVZ). Tangential migration is mediated by the coordination of several chemorepulsive (i.e., sema3A/3F expressed in striatum), permissive (i.e., NRG-CRD highly expressed in GE), and chemoattractive (i.e., NRG1-Ig expressed in cortical VZ/SVZ) substrates which create a corridor along the SVZ of the LGE for migrating interneurons, leading to the formation of defined migratory routes to the cortex. Upon reaching the cortex, interneurons migrate radially within the CP (black arrows) to settle into their final laminar position (mediated by SDF-1/CXCL12 signaling). VZ, ventricular zone; Str, striatum; LGE, lateral ganglionic eminence; MGE, medial ganglionic eminence; Ncx, neocortex. SDF-1/CXCL12, stromal cell derived factor-1/CXC-motif chemokine 12; NRG1, neuregulin-1; Sema3A/3F, semaphorins 3A and 3F.

Ventral telencephalic domains broadly express transcription factors that are crucial to cortical interneuron development (**Figure 2**). Expressed throughout the subpallial subventricular zone (SVZ), the *Dlx* family of homeobox transcription factors is of particular importance for GABAergic interneuron differentiation, migration, and process formation. Specifically, *Dlx1* and *Dlx2* are functionally redundant genes required for GABAergic interneuron production and specification and are also capable of inducing glutamic acid decarboxylase (GAD 65/67) expression in pallial, glutamatergic neuron producing-progenitors (Anderson et al., 1997; Pleasure et al., 2000; Petryniak et al., 2007). Moreover, these genes repress *Olig2*-dependant oligodendrocyte precursor cell (OPC) formation by acting on a common progenitor to determine neuronal versus oligodendroglial cell fate acquisition (Petryniak et al., 2007). *Dlx1/2*-null mutants have a severe deficit in survival and migration resulting in a 70% reduction of these cells in the neocortex (Anderson et al., 1997; Sussel et al., 1999). Working in concert with Dlx1/2, the proneural gene *Mash1* is expressed in the subpallial SVZ and is required for the production and differentiation of GABAergic interneurons (Casarosa et al., 1999; Petryniak et al., 2007; Long et al., 2009). Similar to Dlx1/2, elimination of Mash1 expression results in a substantial decrease in GABAergic neocortical interneurons (Casarosa et al., 1999). While Dlx1/2 and Mash1 are expressed throughout the subpallium, transcription factors that are intimately involved in interneuron fate-specification exhibit a more restricted expression pattern (Flames et al., 2007), raising the possibility that the developing ventral telencephalon contains multiple progenitor pools, each with a distinct progeny fate potential.

### SPATIAL ORIGINS OF INTERNEURON SUBTYPES

The medial ganglionic eminence (MGE) is responsible for the vast majority (~70%) of cortical interneurons (**Figures 1A,B**). Transplantation experiments of MGE precursors have revealed that the majority of MGE-derived interneurons are a heterogeneous group that expresses either PV or SOM (Wichterle et al., 1999; Wichterle et al., 2001; Valcanis and Tan, 2003; Xu et al., 2004). The bulk of this domain expresses the homeobox transcription factor *Nkx2.1*, though the dorsal-most MGE additionally expresses *Nkx6.2* and *Gli1* and is partially *Nkx2.1* negative**(Rallu et al., 2002; Fogarty et al., 2007; Wonders et al., 2008; Sousa et al., 2009). Nkx2.1 is downregulated in neocortical interneuron precursor cells prior to their entry into the cortex, but remains in cells destined for other structures (i.e., the striatum; Marin et al., 2003). *In vivo* loss of function experiments have determined that Nkx2.1 plays a pivotal role in the maintenance and establishment of MGE progenitors as well as the specification of MGE-derived interneuron subtypes located throughout the cortical laminae and that these functions are time dependent (Anderson et al., 2001; Butt et al., 2008). The transcription factor *Sox6* has also been shown to control interneuron subtype differentiation by controlling the temporal segregation of transcriptional programs between progenitors and post-mitotic neurons (Azim et al., 2009; Batista-Brito et al., 2009). Genetic removal of *Sox6* in mice results in failure of MGE-derived interneurons to upregulate PV while SOM expression is largely unaffected (Azim et al., 2009; Batista-Brito et al., 2009). Interestingly, although the affected cells fail to express PV, they are morphologically still like basket cells and continue to exhibit fast-spiking, albeit immature, electrophysiological properties (Batista-Brito et al., 2009).

Several studies have identified multiple subdomains with different expression profiles within the MGE that are responsible for the production of distinct interneuron subtypes (Flames et al., 2007; Fogarty et al., 2007; Wonders et al., 2008). To this point, *in vitro* transplantation studies of dorsal and ventral MGE cultures revealed that, while both regions produce a mixed population of interneurons, there is a strong bias for the production of SOM+ and PV+ cells in the dMGE and vMGE, respectively (Flames et al., 2007; Wonders et al., 2008). In particular, evidence suggests that the Nkx6.2 expressing progenitors in the dMGE preferentially generate SOM-expressing cells and progenitors that express both *Nkx2.1* and *Nkx6.2* are the sole contributor of SOM+/CR+ Martinotti cells in the neocortex (Flames et al., 2007; Fogarty et al., 2007; Sousa et al., 2009). These distinct subdomains within the MGE disproportionately contribute to different areas of the brain. Most notably the majority of globus pallidus neurons, but very few neocortical interneurons, are generated from the most ventral region of the MGE and dorsal PoA (Flandin et al., 2010).

The capability of producing distinct interneuron subtypes both in the MGE and within its subdomains is determined by the coordinated actions of several morphogens, particularly sonic hedgehog (*Shh*) (Sussel et al., 1999; Fuccillo et al., 2004; Xu et al., 2005). Previous studies have shown that Shh maintains Nkx2.1 expression in a Gli3R-independent manner (Gulacsi and Anderson, 2006). Depletion of Shh levels or inhibition of Shh signaling results in a large reduction of detectable Nkx2.1 protein, effectively altering the specification of MGE-derived interneurons in some cases, to a CGE-derived CR+ bipolar fate (Gulacsi and Anderson, 2006; Xu et al., 2010). In addition, higher levels of Shh signaling promote the generation of SOM-expressing interneurons, thereby inversely effecting the number of PV-expressing cells (Wonders et al., 2008). Exposing vMGE progenitors to exogenous Shh in culture can also produce a similar effect on SOM+ fate, suppressing the generation of PV+ interneurons (Xu et al., 2010). Although Shh is secreted from the floor plate of the neural tube causing a low to high dorsal ventral gradient, dorsal MGE progenitors exhibit higher levels of Shh signaling due to enhanced expression of Shh effectors *Gli1* and *Gli2* (Wonders et al., 2008). Hence, these variations in Shh signaling along the dorsal/ventral axis are a potential molecular mechanism for the specification of interneuron subtypes.

While fate mapping more restricted transcriptional domains have revealed a strong bias for the generation of interneuron subtypes, a mixed population of subtypes were still observed (Fogarty et al., 2007). In the dorsal telencephalon, single multipotent progenitor cells exist that are intrinsically capable of generating a diversity of neuronal and glial cell types, though fate-restricted progenitor cells are also present (Luskin, 1998; Shen et al., 2006). Interestingly, specific MGE-derived interneuron subtypes appear to be produced by alternate modes of division. Cell cycle regulators, cyclin D1 and D2 are expressed in distinct progenitor niches, with cyclin D1 predominating in VZ and cyclin D2 in SVZ of cerebral cortex and ganglionic eminences (GE; Glickstein et al., 2007; Glickstein et al., 2009). Cyclin D1 is expressed in both proliferating progenitors and a population of post-mitotic neurons, while cyclin D2 is found only in proliferating progenitors (Glickstein et al., 2007; Koeller et al., 2008). *Cyclin D2*-null mice, exhibit a 30–40% reduction of PV+ interneurons in the neocortex, with no change in the SOM+ interneurons (Glickstein et al., 2007). BrdU birthdating has shown that this depletion occurs throughout gestation, while SOM+ interneuron production is unaffected indicating that this finding was not a result of premature MGE progenitor depletion (Glickstein et al., 2007). Taken together, this data suggests that SOM+ cells are predominantly generated by asymmetric division at the VZ surface, whereas production of the correct number of PV+ neurons may be largely produced by intermediate progenitors in the MGE SVZ (Glickstein et al., 2007).

The next major source of inhibitory neurons is the CGE, which is responsible for producing ~30% of the total cortical interneuron population (**Figures 1A,B**; Anderson et al., 2001; Nery et al., 2002; Miyoshi et al., 2010). Genetic fate-mapping and transplantation experiments have demonstrated that the CGE generates diverse subtypes of interneurons that preferentially inhabit the superficial layers of the neocortex; they are largely marked by the expression of serotonin receptor 3A and include rapidly adapting, CR and/or VIP-expressing, bipolar and double bouquet interneurons as well as LS, reelin expressing neurogliaform cells (**Figure 1C**; Lee et al., 2010; Miyoshi et al., 2010). Although the genetic determinants of MGE-derived cortical interneurons have been characterized over the years, transcription factors specific to the CGE remain elusive. To date, the orphan nuclear receptor, COUP-TFII, has been identified to be widely expressed within CGE and directly involved in the migration and specification of CGE-derived interneurons in the neocortex (Tripodi et al., 2004; Butt et al., 2008; Kanatani et al., 2008; Lodato et al., 2011b). A recent study showed that *Prox1*, a homeobox encoding gene, is expressed in a subset of CGE/LGE- and PoA-derived interneurons during embryonic development and maintained in the mature cortex (Rubin and Kessaris, 2013). Further efforts to identify CGE-specific genes will help elucidate the molecular mechanisms underlying interneuron specification within this spatial domain.

Unlike the MGE and CGE, the contribution of cortical interneurons by the LGE remains controversial. Although some evidence suggests the LGE produces a small population of Sp8-expressing cortical interneurons, the fate of these cells is currently unknown (Anderson et al., 2001; Xu et al., 2004; Yang et al., 2011; Ma et al., 2012). This region remains difficult to study due to its lack of physical border with the CGE and shared molecular characteristics with the dCGE and dMGE.

Recent studies have identified the PoA as a novel source of cortical interneurons (**Figures 1A,B**; Bulfone et al., 1993; Puelles et al., 2000; Cobos et al., 2007; Flames et al., 2007; Gelman et al., 2009). Similar to the MGE, progenitors in this region express *Nkx2.1*, though do not appear to express Lhx6 (Gelman et al., 2009). Because *Nkx2.1* is expressed in both MGE and PoA progenitors, lineage analysis of the PoA is difficult. A recent fate mapping analysis circumvented this issue by using *Nkx5.1*, a transiently expressed transcription factor by early post-mitotic PoA-derived cells, in order to permanently label these cells into adulthood. Here, the PoA was identified as a novel source of a relatively small population of GABAergic cortical interneurons with uniform properties such as rapidly adapting low frequency firing, multipolar morphology, and 30% of which solely express NPY (Gelman et al., 2009).

### TEMPORAL ORIGINS OF INTERNEURON SUBTYPES

Akin to excitatory neurons, cortical interneurons are specified in an “inside-out” manner and this laminar diversity is produced in a temporal sequence (Cavanagh and Parnavelas, 1989; Anderson et al., 2002; Miyoshi et al., 2007). Furthermore, MGE and CGE derived cells are generated with different temporal profiles. While MGE-derived interneurons are mostly born between E11 and E17.5, the majority of CGE-derived interneurons are produced at later developmental time points (E12.5–E18.5, with a peak ~E16.5), and generate distinct interneuron subtypes suggesting time of origin may play a role in determining interneuron specification (Nery et al., 2002; Butt et al., 2005; Miyoshi et al., 2007; Miyoshi et al., 2010; Taniguchi et al., 2012). Both *in vitro* culture assays and fate mapping experiments of temporal cohorts have revealed the competence of MGE progenitors to produce different interneuron subtypes changes over the course of neurogenesis (Xu et al., 2004; Miyoshi et al., 2007). Specifically, a high proportion of SOM+ cells are born at early developmental stages, but are almost absent in E15.5, while PV+ cells are generated at a consistent rate throughout MGE-derived interneuron production. Moreover, each temporal cohort exhibits unique physiological properties characteristic of their birthdate. A particularly interesting example of this temporal diversity phenomenon is Chandelier cells. These neurons typically, though not always, express PV+ and are fast-spiking located both superficially and in the deeper regions of the cortex. Recent efforts elegantly demonstrated that these cells are predominantly produced in the vMGE at the later stages, around E15.5–E17.5, of interneuron production (Inan et al., 2012; Taniguchi et al., 2012).

Contrary to the MGE, interneuron subtypes generated within the CGE appear not to significantly change over time. CGE-derived cells typically inhabit the superficial layers of the neocortex, but there is no correlation between their temporal origin and specific layer destination (Miyoshi et al., 2010). Taken together, this suggests time of origin plays a role in the laminar positioning and specification of interneurons generated in the MGE, but not CGE.

## MIGRATORY MODES OF NEOCORTICAL INTERNEURONS

Neocortical interneurons exhibit a stereotypical organization in the cortical laminae that differs between functionally distinct neocortical regions and is vital for proper patterning of the cortical output. One process believed to be imperative for proper arrangement of neocortical interneurons is migration. Neocortical interneurons generated in the MGE and the PoA undertake complex migratory routes to reach their final destination in the neocortex (**Figure 2**; Corbin et al., 2001; Marin and Rubenstein, 2001, 2003). They migrate tangentially over long distances to enter the neocortex through the marginal zone (MZ) or the intermediate/SVZ before turning radially to reach their absolute location in the neocortex. Similar to excitatory neurons, inhibitory interneurons generated in the MGE and the PoA display birth date-dependent laminar distribution in the neocortex (Ang et al., 2003; Batista-Brito and Fishell, 2009; Miyoshi and Fishell, 2011), thereby arguing for a regulated process of interneuron migration. However, the long-distance tangential migration of interneurons has been considered to be mostly random (Ang et al., 2003; Tanaka et al., 2009). It is unclear how random tangential migration of individual interneurons could lead to an organized distribution pattern in the neocortex for constructing functional circuits, e.g., repetitive columnar microcircuits. In this section, we will review the current literature regarding migration and its role in the spatial organization of distinct interneuron subtypes.

### TANGENTIAL MIGRATION

In contrast to cortical projection neurons, interneurons specified for the neocortex have a distinct and characteristic tangential mode of migration (**Figure 2**; Corbin et al., 2001; Marin and Rubenstein, 2001). These cells cross the cortico-striatal boundary and enter the cortex through two restricted migratory routes: a superficial path within the MZ and a deep route along the SVZ/intermediate zone (IZ; Lavdas et al., 1999). Analogous to their diversity, the migratory trajectories of interneurons display distinct spatial and temporal patterns. At early embryonic stages (<E12 in mice), neocortical interneurons avoid the developing striatum and enter the preplate. At mid-embyonic stages (E12.5–E14.5), cells circumvent the striatal mantle and invade the MZ and IZ/SVZ, avoiding the CP. Finally, in the late stages, they tangentially migrate primarily through the IZ/SVZ route. Once in the cerebral cortex, interneurons disperse tangentially and then generally enter the CP by turning to migrate radially to their final positions (Polleux et al., 2002; Ang et al., 2003; Tanaka et al., 2003; Hevner et al., 2006). Prior to invading the CP, MGE-derived interneurons migrate laterally and disperse widely throughout the cortex whereas CGE-derived interneurons migrate caudally (Yozu et al., 2004).

Little is known about the extracellular substrates guiding interneurons to the MZ or SVZ/IZ migratory routes. Tangentially migrating neurons have been reported to contact corticofugal axons in the IZ and MZ of the developing cortex suggesting that these cells use axons as a scaffold to migrate into the cortex (Metin and Godement, 1996; Parnavelas, 2000). It has been shown that TAG-1 positive corticofugal axons serve as a substrate for neocortical interneurons and blocking TAG-1 function in cortical slices results in reduced migration, though not in *TAG-1*-null mice (Denaxa et al., 2001; Morante-Oria et al., 2003; McManus et al., 2004; Denaxa et al., 2005). Interestingly, early born GABAergic cells colocalize with TAG-1+ axons, while later born cells colocalize with TAG-1 negative/neurofilament+ axons suggesting interneurons may have a stage-dependent substrate preference (McManus et al., 2004).

Tangential migration is mediated by the coordination of several guidance cues that function to both selectively repel and attract cortical interneuron populations (**Figure 2**; Marin and Rubenstein, 2003). Interneurons destined for the cortex express neuropilins 1 and 2 (*Npn1/2*), which are responsive to their repulsive ligands Semaphorin (*Sema 3A/3F*) expressed in the striatum (Marin and Rubenstein, 2001). Conversely, other cues direct migration of cortical interneurons via a chemoattractive effect. Specifically, Neuregulin-1 (NRG1) plays a major role in the guiding of interneuron precursors via the regulation of two different isoforms, soluble NRG1-Ig and membrane bound NRG1-CRD (Flames et al., 2004). These isoforms act as long and short range attractants for tangential migration. NRG1-CRD is highly expressed in the GE creating a permissive effect for migrating interneurons through the SVZ of the LGE. NRG1-Ig is expressed in the cortical VZ/SVZ and is involved in attracting the subventricular stream of tangentially migrating interneurons. The NRG receptor, ErbB4 receptor tyrosine kinase, is expressed in migrating interneurons, particularly in PV+ interneurons (Yau et al., 2003). Mutations in NRG1 or its receptor result not only in failure by interneurons to enter the LGE but also a reduction of interneurons in the cortex (Flames et al., 2004). Both *NRG1* and *ErbB4* have been linked to schizophrenia in humans (Stefansson et al., 2002; Harrison and Law, 2006). The combination of these chemorepulsive, permissive, and chemoattractive substrates, therefore, creates a corridor along the SVZ of the LGE for migrating interneurons, leading to the formation of defined migratory routes to the cortex.

### RADIAL MIGRATION

After entering the cortex, interneurons shift to a radial mode of migration to invade cortical layers and integrate into the cortical circuit (Polleux et al., 2002; Ang et al., 2003; **Figure 2**). Radial glial scaffolds may be involved in the migration of interneurons to their proper location in the CP (Poluch et al., 2008). Recent evidence demonstrates that migrating interneurons and radial glia fibers interact via gap junctions expressing the Cx43 subunit and this interaction mediates the tangential to radial migratory switch indicating that some interneurons may utilize the radial glial scaffold as a means to migrate within the CP (Polleux et al., 2002; Elias et al., 2010).

The timing of the tangential to radial migratory switch is regulated via chemokine signaling. Several groups have shown that stromal-derived factor 1 (SDF-1; also known as CXCL12), plays a key role in this process (Stumm et al., 2003; Tiveron et al., 2006; Lopez-Bendito et al., 2008; Li et al., 2008b). SDF-1 is expressed highly expressed by the meninges in the MZ and intermediate progenitors in the IZ/SVZ of the developing cortex whereas its receptor CXCR4 is expressed in migrating interneurons (Lazarini et al., 2003; Zhu et al., 2004; Tiveron et al., 2006). SDF-1 acts as a chemoattractant for CXCR4 expressing interneurons and this interaction is necessary for normal migration and positioning of interneurons in the neocortex (Stumm et al., 2003; Li et al., 2008b). Indeed, constitutive deletion of CXCR4 resulted in interneuron accumulation in the ventral pallium as well as disorganization of their migratory streams in the cortex. These defects ultimately result in abnormal interneuron lamination in post-natal brains (Tiveron et al., 2006; Lopez-Bendito et al., 2008). SDF-1/CXCR4 signaling is dependent on the age of the interneurons and is lost in neonatal interneurons. This loss of attraction coincides with the timing of radial invasion into the CP by interneurons, suggesting that these two processes are linked. Additionally, this signaling is suggested to be directly involved in defining the SVZ/IZ and the MZ as the main migration paths for interneurons (Tiveron et al., 2006). CXCR7 has also been implicated in the normal migration and lamination of interneurons in the somatosensory cortex, which may imply that various chemokine signaling act regionally biased in controlling the distribution of interneurons in the cortex (Sanchez-Alcaniz et al., 2011; Wang et al., 2011). Prior to the downregulation of signaling, neocortical interneurons remain in the MZ for days actively dispersing throughout the cortex (Tanaka et al., 2009). Some have reported this to be in a cell autonomous fashion, however further evidence will need to be accumulated in order to address the “random walk” behavior.

The cellular and molecular events that direct interneuron positioning to specific cortical layers are poorly understood. Abnormal distribution of interneurons has been observed in Nkx2.1, Lhx6, and Sox6 mutant mice suggesting intrinsic factors govern this process (Alifragis et al., 2004; Butt et al., 2008; Azim et al., 2009; Batista-Brito and Fishell, 2009). However, there is evidence that interneurons receive information to their correct laminar position after arriving in the cortex. About 70% of GABAergic interneurons following the IZ/SVZ stream perform “ventricle-directed migration” where they dive down to the ventricular zone surface of the neocortex, make contact, and pause; they then migrate radially to the CP (Nadarajah and Parnavelas, 2002). This occurs at all stages of corticogenesis by neurons positioned at different depths of the cortical anlage (Nadarajah and Parnavelas, 2002). It has been speculated that these neurons receive some layer information from the VZ surface or developing excitatory cells that is essential for correct positioning and integration in the cortex.

Indeed, several lines of evidence suggest projection neurons of the cortex do play an instructive role in directing interneurons to their proper location. Neocortical interneurons invade the CP only after their projection neuron partners, possibly reflecting a need for signals from appropriately located projection neurons (Lopez-Bendito et al., 2008). In line with this notion, a recent study reported abnormal distribution of PV+ and SOM+ interneurons in *Fezf2*-null mice, which do not have Layer 5 specific subcerebral projection neurons (Lodato et al., 2011a). Together these results suggest a model in which cues provided by projection neurons guide cortical interneurons to their appropriate layer.

## SPATIAL AND FUNCTIONAL ORGANIZATION OF NEOCORTICAL INTERNEURONS

Although the neocortex is composed of functionally distinct areas responsible for different processes, its laminar and columnar organization exhibits an overall uniformity (Felleman and Van Essen, 1991; Mountcastle, 1997). The cytoarchitectonic similarities between regions suggest that there is a “canonical” connectivity between excitatory cells in the same column. Indeed, vertically oriented excitatory neurons are well connected in a direction-selective manner (Weiler et al., 2008; Lefort et al., 2009). Thalamic afferents synapse on to Layer 4 stellate cells, which project to Layer 2/3 pyramidal neurons. These cells project their axons to deeper layers which relay the efferent transmission to other cortical or subcortical regions (Thomson and Bannister, 2003). This activity is shaped in all cortical layers by functionally heterogeneous neocortical interneurons. Despite the importance in regulating excitatory networks, our understanding of the neocortical inhibitory circuitry remains sparse. A number of groups have begun to elucidate the principles governing the organization and wiring of interneurons in the neocortex.

The functional role of distinct interneuron subtypes within the neocortical circuitry is determined by its intrinsic properties and connectivity patterns. Different interneuron subtypes participate in multiple forms of activity such as feedforward or feedback inhibition that modulate the cortical patterning in a variety of ways. PV-expressing cells are almost exclusively fast-spiking cells that synapse onto perisomatic regions, and in the case of Chandelier cells, the axon initial segment of pyramidal neurons. These types of inhibitory connections occur from interneurons that are proximal to their intralaminar excitatory targets, such as Layer 4 fast-spiking basket cells (Dantzker and Callaway, 2000; Yoshimura and Callaway, 2005; Sun et al., 2006). The dendritic network of PV+ cells exhibits varying arbor lengths allowing them to extend across vertically oriented functional columns. In the somatosensory cortex, this is believed to aid in silencing other cortical columns for lateral competition (Adesnik and Scanziani, 2010). Alternatively, SOM-expressing interneurons such as Martinotti cells, are non-fast spiking cells which form connections on the distal dendrites of pyramidal neurons, which results in interlaminar targeting within the cortical column (Silberberg and Markram, 2007; Berger et al., 2009). Moreover, they also form intralaminar connections with nearby pyramidal neurons resulting in disynaptic inhibition contribution to the microcircuitry. These connectivity and firing pattern disparities suggest complementary roles in the cortical circuitry between these subtypes. In general, the high firing frequency and strength of PV+ cells has been found to be imperative for maintaining tight control of gamma rhythms in the brain. On the other hand, SOM+ neurons are slower acting and appear to control the gain of cortical activity. Specifically, recent optogenetic studies have observed that PV+ interneurons sharpen cortical feature selectivity and improve perceptual discrimination via tuning in the V1 of mice (Gee et al., 2012; Lee et al., 2012).

The fine organization and quality of information across cortical domains has been attributed to the highly-ordered arrangement of neocortical neurons and their connections (Michalopoulos and DeFrances, 1997; Mountcastle, 1997; Buxhoeveden and Casanova, 2002; Mountcastle, 2003). Yet some studies have suggested that inhibitory connections to the neocortical circuitry are not specific, and thus interneurons lack selectivity of their targets (Fino and Yuste, 2011; Packer and Yuste, 2011). In particular, experimental evidence suggests Martinotti cells and PV+ interneurons indiscriminately connect to pyramidal cells regardless of their subnetwork affiliation (Fino and Yuste, 2011; Packer and Yuste, 2011). Although distribution of interneuron subtypes varies across cortical regions, these dense connections observed throughout the cortex are believed to be a result of axo-dendritic overlap between projection neurons and interneurons (Packer and Yuste, 2011; Packer et al., 2012). Opposing this notion, a study found that fast-spiking interneurons preferentially connect with neighboring pyramidal neurons that provide them with reciprocal excitation. These pairs also shared common excitatory input and hence belong to the same fine-scale subnetwork (Yoshimura and Callaway, 2005). These inhibitory subnetworks across cortical domains permit the layer-specific coordination of activity in the somatosensory cortex (Adesnik and Scanziani, 2010). Experiments utilizing genetically targeted photostimulation in a mouse knock-in line that conditionally expresses channelrhodopsin-2 in GABAergic neurons revealed that inhibitory connections to excitatory cells exhibit a stereotypic organization that varies between cortical regions suggesting a high-degree of spatial and functional organization of neocortical interneurons (Katzel et al., 2011).

The rich diversity of interneuron subtypes throughout the neocortex allows for almost limitless potential for synaptic connections within functional circuits, yet some studies have shown that interneurons are sparsely connected to one another (Tamas et al., 1998; Gupta et al., 2000; Hughes et al., 2000; Wang et al., 2002). The few interneuron-interneuron synaptic connections identified exhibit a disparity in the strength and number of connections between specific interneuron subtypes. As evidence, PV+ cells form chemical synapses with both PV+ and SOM+ interneurons, however SOM+ cell connections with one another are rarely observed. Such differences have been interpreted to indicate both selectivity and preference in inhibitory neocortical microcircuits (Tamas et al., 1998). A recent study in the mouse visual cortex, however, showed that PV+ interneurons strongly and preferentially inhibit one another compared to other populations, where as SOM+ interneurons avoid inhibiting one another and instead strongly inhibit all other populations. In addition, VIP+ cells preferentially target SOM+ cells (Pfeffer et al., 2013). The functional relevance of the specificity in interneuron connectivity is only beginning to become clear. In the case of VIP+ and SOM+ cells, for instance, a recent study showed that active whisking as well as stimulation of the long-range input into the somatosensory cortex, strongly and specifically recruits VIP-expressing interneurons, which then inhibit SOM-expressing interneurons, and thereby release the inhibitory influence of SOM+ cells on the distal dendrites of connected pyramidal cells (Lee et al., 2013). Further work along this line will help elucidate the underlying logic as well as the functional significance of the diversity in connectivity patterns among molecularly distinct interneuron populations. In addition to chemical synapse-based connectivity, interneurons are intricately connected with one another via electrical gap junctions. This gap junction-mediated electrical coupling is a critical feature of GABAergic interneurons as it allows them to communicate and coordinate their activity within the repetitively organized cortical microcircuitry and thereby generate coherent network activity (Wang and Buzsaki, 1996; Galarreta and Hestrin, 1999; Gibson et al., 1999; Borgers et al., 2005; Hasenstaub et al., 2005). These electrical connections are prominent in 80% of all PV+ interneurons, but are also observed in other interneuron subtypes (Beierlein et al., 2000; Venance et al., 2000; Deans et al., 2001; Galarreta and Hestrin, 2001; Blatow et al., 2003; Chu et al., 2003; Galarreta et al., 2004; Gibson et al., 2005; Ma et al., 2011).

## CLONAL PRODUCTION AND ORGANIZATION OF NEOCORTICAL INTERNEURONS

**FIGURE 3:**
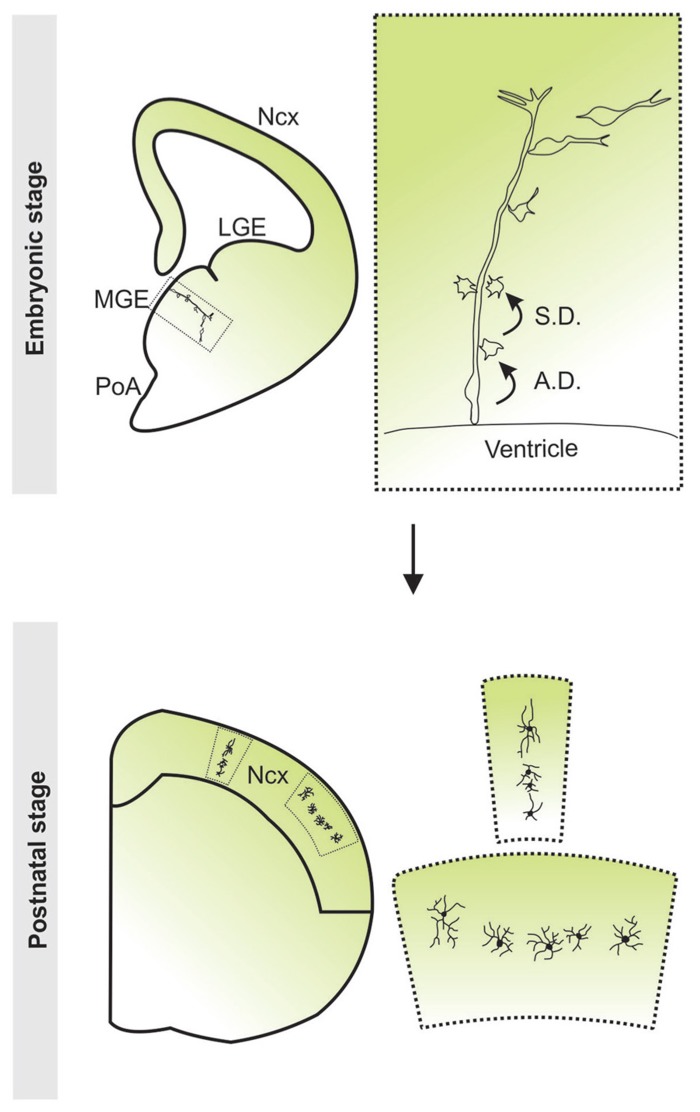
**Clonal production and organization of neocortical interneurons.** Radial glial progenitor cells divide asymmetrically at the ventricular zone surface to self renew****and to simultaneously produce differentiating interneurons or intermediate progenitor cells which****then divide symmetrically in the subventricular zone to produce differentiating interneurons. The progeny of the same radial glial progenitor cell initially migrate along the mother radial glial cell and form radially aligned clonal clusters. As development proceeds, the early-born cells progressively move away from the ventricular zone, acquire the characteristic morphological and biophysical features of differentiating interneurons, and migrate tangentially towards the neocortex. After arriving at their destination in the neocortex, inhibitory interneuron clones do not randomly disperse, but form spatially organized vertical or horizontal clusters. Ncx, neocortex; LGE, lateral ganglionic eminence; MGE, medial ganglionic eminence; PoA, preoptic area.

Similar to the dorsal telencephalon, it was recently discovered that progenitor cells in the ventricular zone of the subpallium are radial glial cells in nature and exhibit morphological traits characteristic of radial glial cells including, a short process extending to the ventricular surface with a large end-foot and a radial process pointing towards the pial surface (Brown et al., 2011). These cells were shown to undergo interkinetic nuclear migration and divided asymmetrically at the ventricular zone surface to self-renew and to simultaneously produce differentiating interneurons or intermediate progenitor cells that migrate away from the VZ. Intermediate progenitor cells further divided symmetrically within the SVZ to produce post-mitotic interneurons. The progressive progeny of an MGE radial glial progenitor closely associated with the radial process of the mother cell, forming a radial clone. During development, the early-born cells progressively moved away from the ventricular zone, acquired the characteristic morphology and biochemical and biophysical properties of differentiating interneurons, and migrated tangentially towards the neocortex (**Figure 3**). Previous reports have shown that cell–cell contact plays an important role throughout neuronal development via transmembrane receptor molecules and local accumulation of secreted signals. This may allow daughter cells to begin neuronal differentiation prior to tangential migration. Some evidence suggests that direct contact with radial glia promotes GABAergic interneuron differentiation (Wu et al., 2008). Moreover, *in vitro* assays have shown newly generated GABAergic neurons acquire excitability more rapidly when cocultured with radial glial cells compared to isolated cultures (Li et al., 2008a). Consistent with this, Brown et al. found that cells within individual clones with the most pronounced neuronal and physiological characteristics are farthest from the ventricular zone (Brown et al., 2011). They possess the typical bipolar morphology of a tangentially migrating interneuron, indicating that they are poised for tangential migration. Cells with less pronounced neuronal characteristics are located close to the ventricular zone. Early electrical activity is characteristic of interneurons shortly after birth and is necessary to propel migration via calcium driven cell motility (Lopez-Bendito et al., 2003; Morante-Oria et al., 2003; Cuzon et al., 2006). It is, therefore, possible that the direct contact between daughter cells and mother radial glial fibers observed by Brown et al. promotes functional development until a cell is mature enough to initiate tangential migration.

Interestingly, in addition to the clonal production of neocortical interneurons in the MGE/PoA, Brown et al. demonstrated that in the mature neocortex, clonally related interneurons do not randomly disperse, but form spatially organized vertical and horizontal clusters (**Figure 3**; Brown et al., 2011). This lineage-dependent clustering of neocortical interneurons has also been shown in a recent study (Ciceri et al., 2013). For this to occur, it is likely necessary for interneuron migration to be tightly regulated at the clonal level. The migratory behavior of interneurons is determined by their response to several extrinsic factors, ultimately establishing their position in the neocortex. The differential interpretation of these signals by an individual interneuron is governed by its expression of various migratory cue receptors. Furthermore, it has been suggested that distinct interneuron subtypes exhibit similar responses to migratory cues (Powell et al., 2001; Powell et al., 2003b). Thus, it is possible that response to migratory cues is designated at birth by a combination of transcription factors and this results in predetermined positioning of interneurons in the neocortex; a potential mechanism for the spatial organization of clonally related interneurons. Alternatively, clones may persistently coordinate their migration while en route to the neocortex. At the cellular level, cell–cell contacts play a role in the regulation of cell motility and are capable of stimulating and inhibiting locomotion in several cell types, though it has yet to be visualized in migrating neocortical interneurons. Indeed, clonally related interneurons were observed to contact one another in slice culture and these interactions influenced their migration (Brown et al., 2011). It is unclear, however, if this behavior plays a role in the clustering of clonal neocortical interneurons. Disruption of these dynamic contacts will need to be performed in order to elucidate any potential involvement in the migration and organization of clonally related interneurons.

The precise generation of diverse subtypes of neocortical interneurons requires intricate regulation of progenitor cell division pattern and dynamics. Brown et al. observed that the MGE and PoA also contain a heterogeneous population of RG cells with an array of proliferative behaviors that can produce a mixed population of PV+ and SOM+ interneurons (Brown et al., 2011). Because different subtypes may express the same neurochemical markers, the authors may have overestimated the amount of fate-restricted progenitors. Future analysis of the morphological and physiological features of interneurons within these clonal clusters will be essential to more thoroughly define the potential of individual MGE progenitors. It is tempting to think that the spatial organization of clonally related interneurons may allow these cells to selectively incorporate into neuronal networks in the neocortex, providing a potential mechanism for specific interneuron connectivity within functional networks.

## CONCLUSION

In this article, we have reviewed recent literature that has helped shape our current understanding of the production, migration, and organization of neocortical interneurons. Similar to excitatory neurons, inhibitory interneurons in the neocortex develop highly specific synaptic connections for the assembly of functional circuits (Gupta et al., 2000; Yoshimura and Callaway, 2005; Thomson and Lamy, 2007; but see Fino and Yuste, 2011). The synaptic connections from local inhibitory interneurons to excitatory neurons exhibit a stereotypic spatial pattern (Katzel et al., 2011), suggesting a spatial and functional organization of neocortical interneurons. The predictable spatial organization of clonally related inhibitory interneurons raises the possibility of a lineage-dependent functional organization of inhibitory interneurons in the mammalian neocortex. Future studies at a single-progenitor resolution will be critical to clarify the basic subtype components of inhibitory interneuron microcircuits in different functional areas of the neocortex, and to ultimately provide insight into the fundamental mechanisms that development uses to construct functional brain circuits.

## Conflict of Interest Statement

The authors declare that the research was conducted in the absence of any commercial or financial relationships that could be construed as a potential conflict of interest.
